# Immunological Adaptations to Pregnancy in Women with Type 1 Diabetes

**DOI:** 10.1038/srep13618

**Published:** 2015-09-22

**Authors:** Bart Groen, Anne-Eva van der Wijk, Paul P. van den Berg, Joop D. Lefrandt, Gerrit van den Berg, Krystina M. Sollie, Paul de Vos, Thera P. Links, Marijke M. Faas

**Affiliations:** 1 Department of Endocrinology, University of Groningen, University Medical Center Groningen, 9700 RB, the Netherlands; 2 Department of Obstetrics and Gynecology, University of Groningen, University Medical Center Groningen, 9700 RB, the Netherlands; 3 Department of Pathology and Medical Biology, Div. of Medical Biology, University of Groningen, University Medical Center Groningen, 9700 RB, the Netherlands; 4 Department of Internal Medicine, University of Groningen, University Medical Center Groningen, 9700 RB, the Netherlands

## Abstract

Despite adequate glycemic control, pregnancy outcome of women with type 1 diabetes (T1D) is still unfavorable as compared to healthy women. In a rat-model of T1D under normoglycemic conditions, adverse pregnancy outcome was also observed, which was associated with aberrant immunological adaptations to pregnancy. Because similar processes may occur in women with T1D we studied the systemic immune response in non-pregnant and pregnant women with and without T1D. The systemic immune response was assessed by using flow cytometry to evaluate the number and activational status of subpopulations of lymphocytes, Natural Killer cells and monocytes in peripheral blood of non-pregnant and pregnant women with and without T1D. An increased white blood cell count, an increased Th1/Th2 ratio, increased Natural Killer cell expression of CD335 and enhanced activation of intermediate and non-classical monocytes was observed in pregnant women with T1D vs. healthy pregnant women. Also, the pregnancy outcome (i.e. incidence of preterm delivery and macrosomia) of women with T1D was unfavorable as compared to healthy women. This study showed that in T1D, the immunological adaptations to pregnancy are disturbed. In addition to hyperglycemia, these different immunological adaptations may be responsible for the greater frequency of complications in pregnant women with T1D.

Despite tight glycemic control, frequent episodes of hyperglycemia still occur during pregnancy in type 1 diabetic (T1D) patients^1^. Up to now, these hyperglycemic episodes have been thought to be responsible for the increased incidence of pregnancy complications^2^. However, recent work suggests that more factors may be involved^3^. We have shown that BBDP-rats, a rat-model of T1D, undergo aberrant immunological adaptations during pregnancy, which are associated with pregnancy complications^3^. Similar events may occur in autoimmune T1D pregnant women.

Normal pregnancy is accompanied by various immunological adaptations facilitating implantation, placentation and tolerance of the semi-allogeneic fetus^4^,^5^,^6^; aberrations in these adaptations are associated with pregnancy complications, like miscarriages, preeclampsia and preterm delivery^7^,^8^,^9^. Normal systemic adaptations to pregnancy include a shift towards a type 2 immune response in T-lymphocytes^4^,^10^ and Natural Killer (NK) cells^11^,^12^ and an increased frequency of regulatory T-cells (Treg) during the first and second trimester of pregnancy^10^,^11^. Moreover, normal pregnancy is accompanied by a generalized activation of the inflammatory response, characterized by activation of monocytes and granulocytes, showing increased expression of various activation markers and altered cytokine production^13^,^14^. The proinflammatory condition of pregnancy is further supported by the fact that an increased amount of intermediate monocytes are found in pregnancy. Intermediate monocytes are a separate monocyte subset, a transitional state between classical and non-classical monocytes, of which the function is not exactly known yet^15^. However, their numbers are increased in pro-inflammatory conditions, such as preeclampsia and other inflammatory diseases^15^,^16^. It is unknown which immunological adaptations to pregnancy occur in women with autoimmune T1D.

The immune response in T1D patients is characterized by a type 1 immune response^17^, impaired function of regulatory T-lymphocytes^18^ and increased mRNA expression of interferon-gamma (IFN-γ)^19^, when compared to healthy individuals. In view of this different immune response in T1D women, we hypothesized that pregnancy-induced immune adaptations differ between T1D and healthy pregnant women. The aim of this study was therefore to assess the systemic immunological adaptations to pregnancy in women with T1D. Understanding these aberrant changes may be a first step toward designing new means, in addition to glycemic control, for preventing pregnancy complications in T1D pregnant women.

## Materials and Methods

### Patients

This study was approved by and carried out in accordance with the guidelines of the ethic committees of the University Medical Center Groningen (UMCG) and Martini Hospital Groningen (NL30779.042.09) and registered at the Dutch trial register (NTR2195). All participants provided informed consent.

Four groups of women (age 18–40 years) were included in the study: healthy non-pregnant (n = 16), healthy pregnant (n = 19), non-pregnant with T1D (n = 19) and pregnant with T1D (n = 21). Healthy non-pregnant women were recruited from personnel at the UMCG and healthy pregnant women from the midwifery clinic. Non-pregnant and pregnant women with T1D were recruited from the diabetes outpatient clinics of the UMCG and the Martini Hospital Groningen. Non-pregnant women filled in a questionnaire to obtain information about their cycle, contraception and last menstrual bleeding. Blood samples were obtained by venous punction into a 10 ml EDTA tube (BD-Plymouth, UK) from non-pregnant women within 10 days after the start of the last cycle and from pregnant women at 30 weeks of gestation. All pregnant women were followed up during pregnancy and 6 weeks postpartum to evaluate pregnancy outcome. For the healthy pregnant women, exclusion criteria were gestational diabetes mellitus, known autoimmune, cardiovascular or other active diseases, >2 previous miscarriages, maternal/fetal complications during pregnancy (i.e. preeclampsia/HELLP, intra-uterine growth restriction, prematurity, perinatal death, congenital malformations and neonatal death). For the healthy non-pregnant women, exclusion criteria were known active diseases. For both diabetic groups, exclusion criteria were HbA1c >7.5% (at the time of sampling), renal failure (serum creatinine >120 μmol/l) and active treatment for autoimmune disease.

### Outcome measures

The basic characteristics and pregnancy outcome variables are shown in Table 1.

### Sample processing

#### Human blood leukocyte counts

100 μl of blood was used to measure total white blood cell numbers (WBC), using a microcell counter (Sysmex PocH 100i, Sysmex, Etten-Leur, The Netherlands).

#### Reagents

Washing buffer (phosphate-buffered saline (PBS) with 0.5% bovine serum albumin and 0.1% NaN3), FACS-TM lysing buffer solution (BD Biosciences, Breda, the Netherlands), FoxP3-staining buffer set (eBioscience, Vienna, Austria) and complete RPMI-1640 medium (Lonza Benelux, Breda, the Netherlands) supplemented with 60 μg/ml gentamycin (Invitrogen, Breda, the Netherlands).

#### Antibodies

We used antibody cocktails for T-lymphoyctes, NK cells. Unless stated otherwise, they were purchased from BioLegend (BioLegend Europe, Uithoorn, the Netherlands):

T-lymphocytes antibody cocktail. PerCP labeled anti-human CD3 (clone UCHT1), APC/Cy7 labeled anti-human CD4 (clone OKT4), PE/Cy7 labeled anti-human CD25 (clone BC96), Alexa Fluor 488 labeled anti-mouse/rat/human FOXP3 (clone 150D), Alexa Fluor 488 labeled mouse IgG1 isotype control (clone MOPC-21), PE labeled anti-mouse/human Tbet (clone eBio4B10, eBioscience, Germany), PE labeled mouse IgG1 isotype control (clone MOPC-21), Alexa Fluor 647 anti-mouse/human GATA-3 (clone TWAJ, eBioscience) and Alexa Fluor 647 mouse IgG1 isotype control (MOPC-21).

Natural Killer cells antibody cocktail. PerCP labeled anti-human CD3 (clone UCHT1), APC labeled anti-human CD56 (clone MEM188), Pacific Blue labeled anti-human CD16 (clone eBioCB16, eBioscience) and PE labeled anti-human CD335 (clone 9E2), PE labeled mouse IgG1 isotype control (clone MOPC-21).

Monocytes antibody cocktail. PerCP/Cy5.5 labeled anti-human CD14 (clone HCD14), Pacific Blue labeled anti-human CD16 (clone eBioCB16, eBioscience), FITC labeled mouse anti-human HLA-DR, DP, DQ (i.e. MHC-II; clone Tu39, BD Pharmingen, Breda, Netherlands), PE labeled anti-human CD62L (clone DREG-56) and PE labeled mouse IgG1 isotype control (clone MOPC-21).

#### Sample labeling

We used antibody cocktails for T-lymphocytes, NK-cells and monocytes. Immediately after sampling, whole blood was mixed with RPMI-1640 (1:1) and aliquoted, centrifuged and aspirated. Subsequently, all tubes were incubated with different antibody-cocktails in the dark for 30 min and washed with 500 μl washing buffer, followed by red blood cells lysis with lysing buffer in the dark for 30 minutes. After centrifugation, aspiration and washing, tubes for lymphocyte/lymphocyte isotype were incubated with fixperm (from FoxP3-staining buffer set) in the dark for 30 minutes. To the other tubes, 300 μl washing-buffer was added and stored. After 30 min incubation in the dark, tubes for lymphocyte and lymphocyte isotype staining were washed with perm (from FoxP3-staining buffer set) followed by incubation with the FoxP3, Tbet and GATA-3 antibodies or their isotype controls for 30 min in the dark at RT and washed with perm. Finally, 300 μl washing buffer was added to these tubes. All tubes were stored in the dark at 4 °C until measurement by flow cytometry within 24hr.

### Flow cytometry

Cells were analyzed using the BD LSR-II flow cytometer (BD Biosciences, Breda, the Netherlands). At least two hundred fifty thousand events were measured and saved. Analysis was performed using FlowJo 7.6.1 (Tree Star, Inc., Ashland, OR, USA).

#### Data analysis

**Figure 1:**
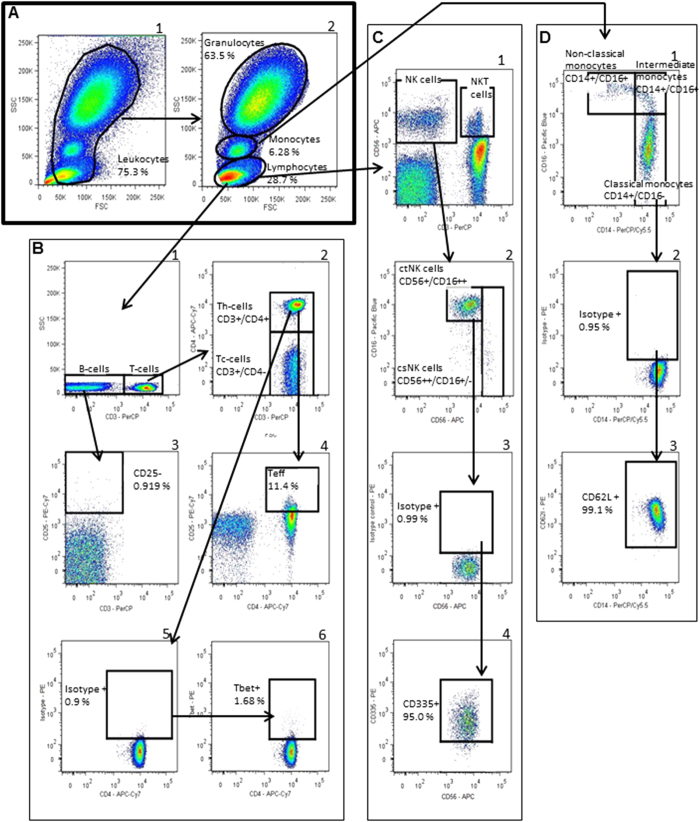
The gating strategy of T-lymphocytes, NK-cells and monocytes. **[Panel-A]:** First a FSC/SSC-plot was made and we gated all leukocytes (A1). Leukocytes were copied to a new scatterplot identifying lymphocytes (including NK-cells), monocytes and granulocytes (A2). **[Panel-B]:** The lymphocyte population was copied to a SSC/CD3-scatterplot identifying T-cells (CD3+) (B1). The T-cell gate was copied to a CD3/CD4-scatterplot identifying Th-cells (CD3+/CD4+) and Tc-cells (CD3+/CD4-) (B2). To identify CD25+ cells (Teff and Treg), we used CD3-cells (i.e. B-cells, which are CD25-negative), to set the gate. Therefore, the CD3- cells were copied to a CD25/CD3-scatterplot and a gate was set in such a way that CD3-cells were >99% negative for CD25 (B3). This gate was copied to a new CD25/CD3-scatterplot in which we copied the Th-cells (B4). Teff-cells were defined as CD25+(B4). The Th-cell gate was copied to a CD4/Tbet-scatterplot identifying type 1 Th-cells (Th1) (B5-6)). We used the isotype for Tbet to define Tbet+ cells. Therefore, in the isotype-control, gates for Tbet were set in such a way that the positive gate contained no more than 1% of the population (B5). This gate was copied to the sample containing the Tbet-antibody calculating percentage of Tbet + Th-cells. Similar gating procedures with isotype controls were used identifying Th2 (CD3+/CD4+/GATA-3+) and Treg (Cd3+/CD4+/CD25+/FoxP3+). **[Panel-C]:** To identify NK-cells, lymphocytes were copied to a CD3/CD56-plot and a gate was set around the CD3−/CD56+ cells and around CD3+/CD56+cells identifying total NK-cell and NKT-cell populations, respectively (C1). The NK-cell gate was copied to a CD56/CD16-scatterplot (C2). CD56dimCD16+ cells were defined as cytotoxic NK (ctNK) cells and CD56brightCD16+/− as cytokine-secreting NK-cells (csNK). To identify expression of CD335 on ctNK-cells, isotype was used as described before (C3-4). Similarly, the CD335-expression on csNK-cells was evaluated. **[Panel D]:** To identify monocytes subsets and activation status, monocytes were copied to a CD14/CD16-plot. Monocytes were divided into three populations, i.e. 1) classical monocytes (CD14++/CD16−), 2) intermediate monocytes (CD14++/CD16+) and 3) nonclassical monocytes (CD14+/CD16+) (D1). To evaluate the activation status of these monocyte subsets, we used MHC-II and CD62L. We used isotypes for MHC-II and CD62L as described before (example of CD62L-expression of classical monocytes in panel D2–3).

Differential cell counts. First, different leukocyte populations (i.e. lymphocytes, monocytes and granulocytes) were assessed, using a forward-/sideward scatterplot (FSC/SSC) of the tube stained for NK-cells. A gate was set around the leukocytes and secondary gates were set around the three different subpopulations in a new FSC/SSC plot (Fig. 1, panel A).

Lymphocytes, NK cells and monocytes. The gating strategy of lymphocytes, NK-cells and monocytes was shown in Fig. 1, panels B–D respectively.

**Figure 2:**
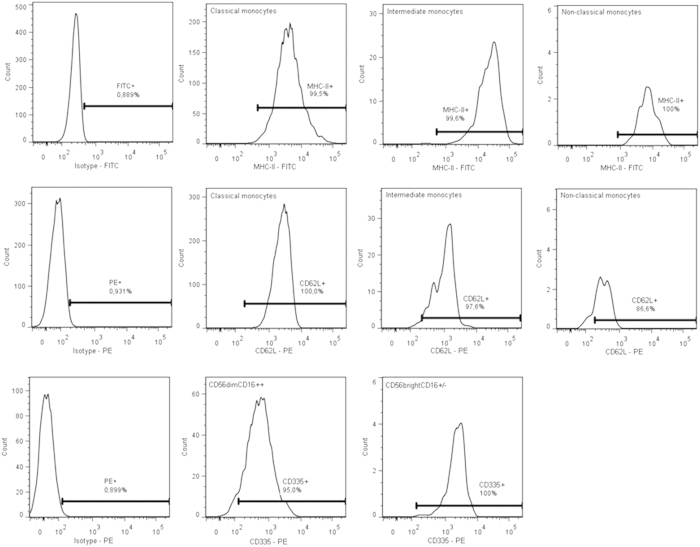
Mean Fluorescent Intensity (MFI) of monocyte MHC II (panel A), monocyte CD62L (panel B) and NK cell CD335 (panel **C**) expression. The left graph in each panel represents the MFI of the isotype control (which was similar for all subtypes). Subpopulations were gated using the gating strategy of Fig. 1. Gates were set in the isotype control in such a way that <1% of the cells stained for the isotype control were positive (percentage positive cells are shown in the graphs). This gate was then copied to the samples containing the antibodies.

Examples of Mean Fluorescence Intensity (MFI) of monocyte MHC-II, CD62L and NK-cell CD335 expression plots are shown in Fig. 2.

### Statistics

Continuous parameters were expressed as mean ± SEM in case of normally distributed data, or as median (Q1–Q3) in case of skewed distribution. Independent T-tests and Chi-square-tests were used for categorical variables (corrected for multiple testing). Two-way ANOVA (with interaction effects) was used to analyze diabetes- and/or pregnancy-induced differences in immune cells. Before performing this two-way ANOVA, normality of the data was tested using the Kolmogorov-Smirnov test. If data were distributed abnormally, log-transformation was used. For all analysis, p-values < 0.05 were considered as statistically significant and p-values between 0.05-0.1 were considered as a statistical trend. Statistical analyses were performed using SPSS for windows version 20.0.0.1 (SPSS, Inc., Chicago, IL).

## Results

### Basic characteristics

The mean age, BMI and smoking status were similar in all 4 groups (Table 1). Mean HbA1c was decreased in pregnant women with T1D as compared to non-pregnant women with T1D. In pregnant women with diabetes, we observed a significantly higher incidence of prematurity, macrosomia and preeclampsia (not significant) as compared to healthy women.

### Peripheral immune response

#### WBC: T1D pregnant patients showed increased WBC-counts

**Figure 3:**
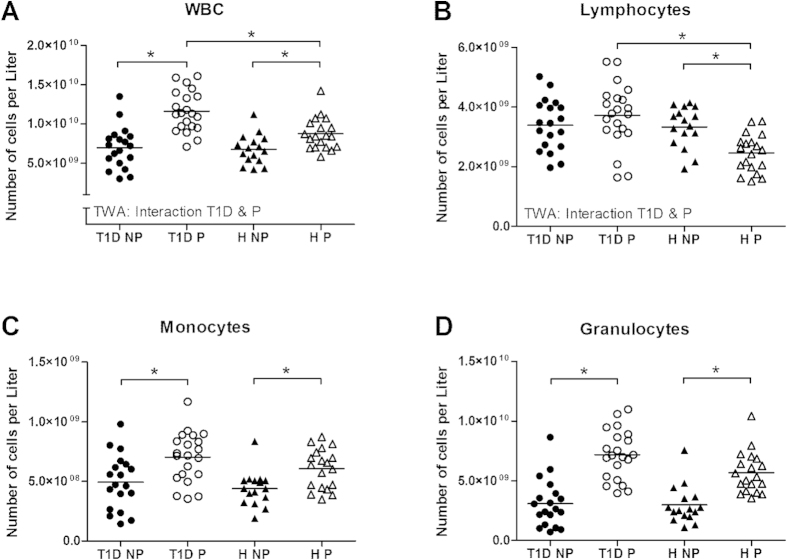
WBC (A), lymphocytes (B), monocytes (C) and granulocytes (D) in non-pregnant (NP) and pregnant (P) women with type 1 diabetes (dotted line) and healthy women (continuous line). *Significant difference between the marked groups, independent T-test, p < 0.05. I: Significant interaction between diabetes and pregnancy, p < 0.05, two-way ANOVA.

WBC-count increased significantly in both diabetic and healthy women during pregnancy (Fig. 3A). However, WBC-count was significantly higher in diabetic pregnant women vs. healthy pregnant women (interaction diabetes and pregnancy; p = 0.018). The number of lymphocytes did not change during pregnancy in women with T1D, whereas a decreased lymphocyte number was found in healthy pregnant woman vs. their non-pregnant controls (Fig. 3B). The number of monocytes and granulocytes was increased in both groups during pregnancy (Fig. 3C,D).

#### Lymphocytes: Increased Th1/Th2-ratio in pregnant women with T1D

**Figure 4:**
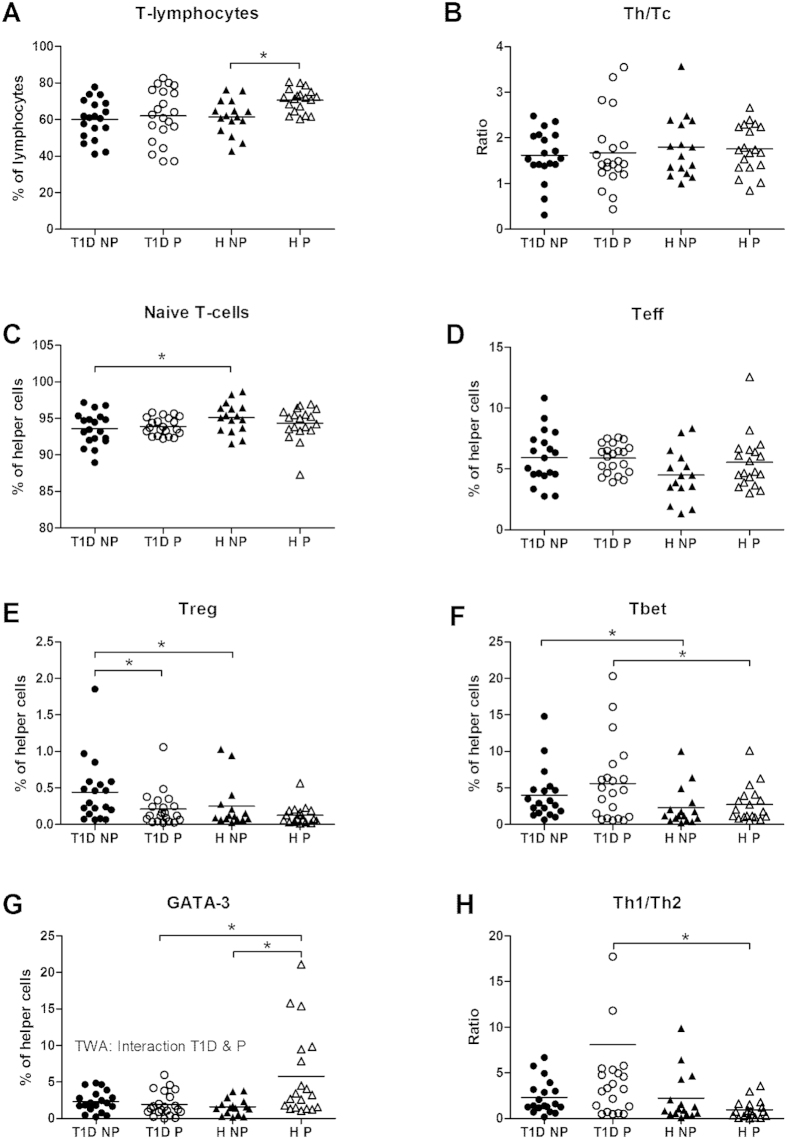
The percentage of T-lymphocytes (A), ratio of helper (Th) and cytotoxic (Tc) T-cells (B), percentage of naive T-cells (C), effector T-cells (D), regulatory T-cells (Treg) (E), Tbet positive Th (as a transcription factor for Th1) (F), GATA-3 positive Th (as a transcription factor for Th2) (G) and the ratio Th1/Th2 (H) in non-pregnant (NP) and pregnant (P) women with type 1 diabetes and healthy women. *Significant difference between the marked groups, independent T-test, p < 0.05. I: Significant interaction between diabetes and pregnancy, p < 0.05, two-way ANOVA.

The percentage of T-lymphocytes was similar in non-pregnant diabetic patients and healthy women (Fig. 4A). During pregnancy, the percentage of T-lymphocytes increased in healthy women only. The ratio of helper T- (Th) and cytotoxic T-cells (Tc) and the number of effector T-cells (Teff) cells were not affected by either diabetes or pregnancy (Fig. 4B,D). The percentage of CD4+CD25- was lower, while the percentage of CD4+CD25+FoxP3+ cells was higher in non-pregnant diabetic vs. non-pregnant healthy women; these differences were not observed during pregnancy (Fig. 4C,E). The percentage of Tbet positive Th cells was significantly higher in both non-pregnant and pregnant diabetic women as compared to their respective healthy controls (Fig. 4F). The percentage of GATA-3 positive Th-cells was similar in the non-pregnant groups, while it was increased in healthy pregnant women only (Fig. 4G). This resulted in an increased Th1/Th2 ratio (p = 0.013) in pregnant women with diabetes vs. healthy pregnant controls (Fig. 4H).

#### NK-cells: Increased expression of the activating NK cell receptor NKp46 on NK-cell subsets in pregnant women with T1D

**Figure 5:**
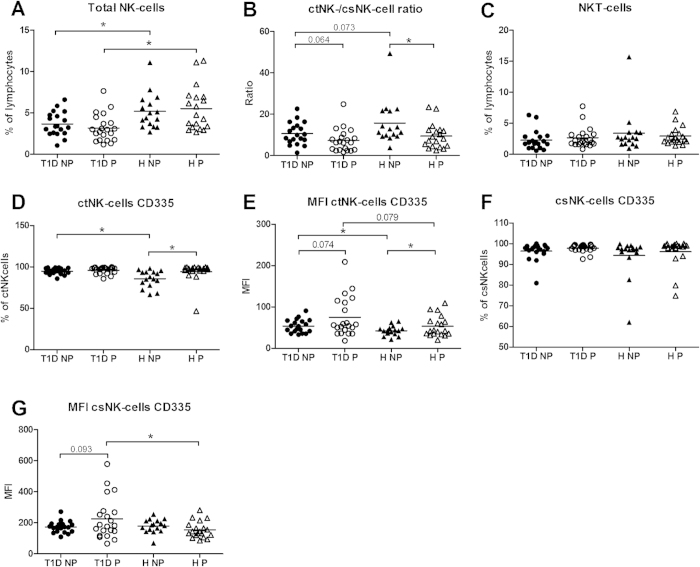
The percentage of total NK-cells (A), ratio of cytotoxic (ct)NK-cells and cytokine secreting (cs)NK-cells (B), NKT-cells (C), the percentages and the mean fluorescence intensity (MFI) of CD335 on ctNK (D,E) and csNK-cells (F,G) in non-pregnant (NP) and pregnant (P) women with type 1 diabetes and healthy women. *Significant difference between the marked groups, independent T-test, p < 0.05.

The percentage of total NK-cells was lower in pregnant and non-pregnant women with diabetes than in the respective healthy controls (Fig. 5A). A trend towards a decreased ratio of cytotoxic NK- (ctNK) and cytokine secreting NK-cells (csNK) was observed in non-pregnant women with T1D as compared to healthy non-pregnant women (Fig. 5B). During pregnancy, this ratio decreased in healthy women and tended to decrease in diabetic women. The percentage of NKT-cells was influenced neither by pregnancy nor diabetes (Fig. 5C).

To show functional changes of NK cells, we studied the expression of the activating receptor NKp46 (CD335). We choose to study this receptor, since studies have shown that the expression of NKp46 may not only be associated with diabetes development^20^, but also with infertility^21^ and preeclampsia^22^.

Most of the ctNK-cells were positive for CD335 (>80%). The percentage of positive cells and the MFI of CD335 for ctNK-cells was significantly higher in non-pregnant diabetic women than in non-pregnant healthy women (Fig. 5D,E). During normal pregnancy, both the numbers and the MFI of CD335+ ctNK-cells increased as compared to non-pregnant controls. In T1D, we only observed a trend towards an increased MFI of CD335.

Almost all csNK-cells expressed CD335; the percentage of positive cells was not affected by diabetes or pregnancy (Fig. 5F). The MFI of CD335 on csNK-cells was similar in both non-pregnant groups (Fig. 5G). However, we observed an increased (trend) MFI in pregnant diabetic women as compared to non-pregnant diabetic women. This resulted in a significantly increased MFI of CD335 in pregnant women with T1D vs. healthy pregnant women.

#### Monocytes: Increased activation of intermediate and non-classical monocytes in pregnant women with T1D

**Figure 6:**
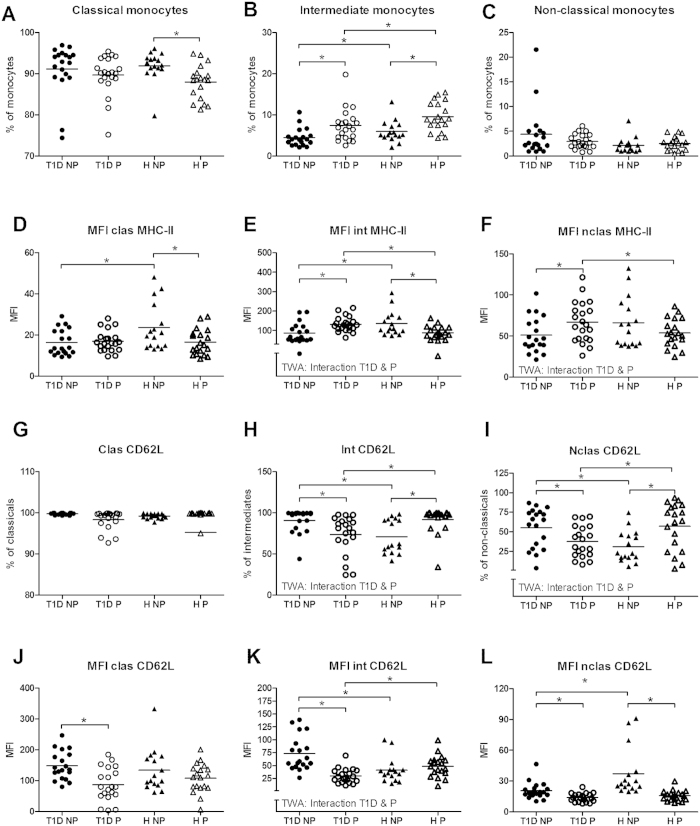
The percentage of classical, intermediate and non-classical monocytes (**A–C**), MFI of MHC-II of the three subsets (D–F), and percentage and MFI of CD62L positive monocyte subsets (G–L) in non-pregnant (NP) and pregnant (P) women with type 1 diabetes and healthy women. *Significant difference between the marked groups, independent T-test, p < 0.05. I: Significant interaction between diabetes and pregnancy, p < 0.05, two-way ANOVA.

The percentage of intermediate monocytes was decreased in non-pregnant women with diabetes as compared to healthy non-pregnant women, while percentages of classical and non-classical monocytes did not differ between these 2 groups (Fig. 6A–C). In healthy pregnant women we found a decreased percentage of classical monocytes and an increased percentage of intermediate monocytes vs. healthy non-pregnant women. In women with diabetes, only an increased percentage of intermediate monocytes was found during pregnancy as compared with non-pregnant women.

In all groups of women, the three monocyte subsets were 100% positive for MHC-II (data not shown); however, the MFI of MHC-II differed between the subsets (being lowest in classical monocytes and highest in intermediate monocytes) and was affected by diabetes and pregnancy (Fig. 6D–L). In non-pregnant diabetic women, the MFI of MHC-II of classical and intermediate subsets was significantly lower as compared with healthy non-pregnant controls. In healthy pregnant women, MFI of MHC-II of classical and intermediate monocytes significantly decreased as compared to non-pregnant women. In contrast, in pregnant women with T1D, MFI of MHC-II significantly increased in intermediate and non-classical monocytes, resulting in an increased MFI of MHC-II of intermediate and non-classical monocytes in pregnant women with T1D vs. healthy pregnant women.

In all four groups, almost all classical monocytes were positive for CD62L. This was not the case for the intermediate and non-classical subsets. In these subsets the percentage of CD62L positive monocytes was higher in non-pregnant women with diabetes vs. healthy non-pregnant controls, while the opposite was observed during pregnancy (interaction pregnancy and diabetes; p = 0.00006 and p = 0.0003). In non-pregnant women, the MFI of CD62L on classical monocytes was not affected by diabetes, while this MFI was increased in intermediate monocytes and decreased in non-classical monocytes in women with diabetes as compared with healthy women. During healthy pregnancy, MFI of CD62L was not affected in classical and intermediate monocytes, but was decreased in non-classical monocytes. By contrast, MFI of CD62L was decreased in all monocyte subsets during a diabetic pregnancy.

## Discussion

This is the first study showing differences in the systemic immune response in pregnant women with T1D as compared with healthy pregnant women. The strength of our study is that we evaluated various subpopulations of immune cells, of the specific and the innate arm, and their activation status, giving us a broad overview of changes in different cells populations. Our data showed that pregnant women with T1D have an increased WBC-count, Th1/Th2 ratio, and expression of NKp46 on NK-cells and enhanced activation of intermediate and non-classical monocytes as compared to healthy controls. The exact mechanisms are unclear and probably multifactorial. The immunological adaptations in normal pregnancy are suggested to be driven by factors such as increased sex hormone concentrations^23^, presence of the placenta^24^ and the production of factors by the placenta, like placental microparticles or cytokines^25^. Aberrant adaptations in diabetic pregnancy may be due to changes in either of these factors. Alternatively, it may also be due to the presence of autoimmune disease, inducing differences in immune cells, resulting in a different response to pregnancy factors.

It is well known that the immune response of non-pregnant diabetic patients differs from that of healthy subjects^17^,^18^,^19^,^26^,^27^. Our data confirmed an increased Th1 response and an increased activation status of monocytes in diabetic women vs. healthy women^26^,^27^,^28^. Moreover, we augment previous knowledge by showing that only intermediate and non-classical monocytes are activated. We also showed an increased percentage of Treg in non-pregnant diabetic women, which may act as a compensatory mechanism for impaired Treg function^18^. Moreover, we found decreased NK-cell numbers in non-pregnant diabetic women, which may be related to this defective Treg function^29^. CD335 (or NKp46) , a member of the cytotoxicity receptor-family^30^, is essential in the development of T1D^20^. This may explain why this receptor is upregulated on ctNK-cells in non-pregnant diabetic women.

Various studies observed a decreased Th1/Th2 ratio during healthy pregnancy^4^,^10^. Although we did not find this, we observed an increase in GATA-3 positive Th lymphocytes in healthy pregnant women. The reason for this discrepancy is not clear, but may be due to methodological differences^31^. The decreased percentage of Treg during pregnancy is in line with earlier reports^32^,^33^. This decrease in Treg appeared to be more pronounced in diabetic women, suggesting that Treg do adapt to a diabetic pregnancy. The increase in Th1 response in diabetic pregnancy was also found in non-pregnant diabetic women, suggesting that this is due to the diabetic state. We observed an increased Th1/Th2 ratio in diabetic pregnancy. As such an increased ratio is associated with pregnancy complications, such as preeclampsia and miscarriage^7^,^10^, it may be suggested that the increased Th1/Th2 ratio in diabetic pregnancy is be involved in the increased numbers of pregnancy complications in this condition.

In contrast to other studies, pregnancy did not affect the total percentage of NK-cells in healthy controls^11^,^34^. However, in line with former studies, the absolute numbers of NK-cells tended to decrease in healthy pregnancy (1.73·10^8^ vs. 1.28·10^8^, p = 0.053), but not in T1D pregnancy (1.21·10^8^ vs. 1.12·10^8^, p = 0.652). This may be due to the already low frequency of these cells in non-pregnant diabetic women; a further decrease may not be required during pregnancy. We observed increased CD335 expression on both NK-cell subpopulations in pregnant women with diabetes. The expression of CD335 (or NKp46) is negatively correlated between csNK cell CD335 expression and type 1 cytokines, such as TNF-α^21^,^35^ in a fertile population, but not in women with recurrent pregnancy loss or implantation failure^21^. This may suggest that signal transduction via CD335 on NK cells is changed in these pregnancy complications. If this is also the case in diabetic pregnancy, upregulation of CD335 may be a compensatory mechanisms.

We found increased expression of MHC-II and decreased expression of CD62L on intermediate monocytes in diabetic pregnant women, suggesting activation of these cells. Decreased MHC-II expression on monocytes is associated with inadequate presentation of antigens to T-cells and disturbed T-cell stimulation^36^. Thus, the ability of these monocytes to present antigens to T-cells is increased in pregnant women with diabetes, which could lead to enhanced activation of the systemic immune response. Shedding of CD62L is usually associated with the activation of monocytes^37^, indicating that the monocytes of diabetic women may be further activated during pregnancy. Intermediate monocytes have been suggested to play a role in the pathophysiology of preeclampsia^16^,^38^. It may therefore be suggested that increased activation of intermediate monocytes during diabetic pregnancy, may be involved in the increased incidence of preeclampsia in these women.

Our observations of aberrant immunological changes in diabetic pregnancy raise further questions. Various adaptations of the immune response are required for adequate implantation, placentation and tolerance of the semi-allogeneic fetus^4^,^5^,^6^. We hypothesize that the increased pregnancy complications in diabetic pregnancy are partly the result of the disturbed immunological adaptations, immunological maladaptations may lead to pregnancy complications^7^,^8^,^9^. We cannot completely rule out a role for hyperglycemia, since, although the mean HbA1c was significant decreased in pregnant T1D women as compared to non-pregnant T1D women (which is most likely the result of the preconceptional care of these women^39^), it is still increased as compared to healthy pregnant women (mean HbA1c 4.7% for healthy pregnant women with a gestational age of 30 weeks^40^). However, our suggestion of immunological involvement is corroborated by our findings in an animal model for T1D, in. In this model, in the face of normoglycemia, similar immunological maladaptations as in the present study, were associated with increased pregnancy complications^3^. Our hypothesis is further endorsed by the fact that in the present study women with T1D and complicated pregnancy outcome (i.e. preeclampsia or preterm delivery) showed even higher Th1/Th2-ratios and more pronounced activation of monocytes as compared to diabetic women with normal pregnancy outcome (data not shown). Moreover, our suggestion that disturbed immunological adaptations in an autoimmune environment contributes to pregnancy complications is in line with findings in other autoimmune diseases, such as rheumatoid arthritis or systemic lupus erythematosus^41^,^42^. Unfortunately, due to the relatively small sample size in this study, we were not able to correlate immunological parameters to pregnancy outcome parameters.

In conclusion, this study showed that in T1D the pregnancy induced immunological adaptations are disturbed. Importantly, these different immunological adaptations may be responsible, as also shown for other autoimmune diseases, for the increased frequency of complications in pregnant women with T1D. Further mechanistical insights into the role of various immune cells in implantation as well as placental and fetal development in healthy and diabetic pregnancy are warranted and may lead to new therapeutic approaches to improve pregnancy outcome of women with T1D.

## Additional Information

**How to cite this article**: Groen, B. *et al.* Immunological Adaptations to Pregnancy in Women with Type 1 Diabetes. *Sci. Rep.* **5**, 13618; doi: 10.1038/srep13618 (2015).

## Figures and Tables

**Table 1:** Basic characteristics of the study groups.

Basic characteristics	Type 1 diabetesNP (n = 19)	Type 1 diabetesP (n = 21)	Healthy NP (n = 16)	Healthy P (n = 19)
Age (yrs)	30.2 ± 1.2	28.8 ± 0.8	27.5 ± 1.2	29.6 ± 1.1
Body Mass Index (kg/m2)	24.2 (21.4–28.7)	23.7 (20.6–26.6)	21.8 (20.3–26.1)	22.5 (20.3–23.3)
Smoking (n)	3 (15.8%)	0 (0%)	1 (6.3%)	0 (0%)
Alcohol use (n)	12 (53.2%)	0 (0%)*	14 (87.5%)	0 (0%)*
Duration of diabetes	17.0 ± 2.0	15.2 ± 1.9	n/a	n/a
Max. insulin dosage (IU/24 h)	50 ± 14	67 ± 31	n/a	n/a
Mean HbA1c% (mmol/mol)	7.7 ± 0.2 (60 ± 2)	6.5 ± 0.1* (48 ± 1)	n/a	n/a
Proteinuria (n)	0 (0%)	0 (0%)	0 (0%)	0 (0%)
Nulliparous (n)	11 (57.9%)	12 (57.1%)	13 (81.3%)	12 (63.2%)
DA at sampling (wks)	n/a	31 ± 2†	n/a	30 ± 1
GA at delivery (wks)	n/a	37 ± 1†	n/a	40 ± 1
PE/HELLP (n)	n/a	2 (9.5%)	n/a	0 (0%)
Prematurity (n)	n/a	5 (23.8%)†	n/a	0 (0%)
Caesarean Section (n)	n/a	6 (28.6%)	n/a	2 (10.5%)
Perinatal Mortality (n)	n/a	0 (0%)	n/a	0 (0%)
Macrosomia (n)	n/a	11 (52.4%)†	n/a	1 (5.3%)
Birth weight	n/a	3646 ± 126	n/a	3586 ± 88

Values are shown as mean ± SEM, except for BMI, which was shown as median (Q_1_–Q_3_), and as numbers (percentage). NP: non-pregnant, P: pregnant, DA: duration of amenorrhea, GA: gestational age.

Definitions of different outcome variables: ‘smoking’ (yes in case of smoking ≥1 cigarette/day and no in case of non-smokers or women who stopped smoking after a positive pregnancy test), ‘alcohol use’ (yes in case of drinking ≥1 unit/day and no in case of non-drinkers or women who stopped after a positive pregnancy test), Maximal dosage of insulin (IU) per 24 h at moment of sampling in type 1 diabetes NP and during pregnancy in type 1 diabetes P. ‘HbA1c’ (for non-pregnant women with diabetes: mean HbA1c of the last year; for pregnant women with diabetes: mean HbA1c during pregnancy), ‘proteinuria’ (yes in case of albuminuria >16.0 mg/l at day of sampling for non-pregnant women and at beginning of pregnancy for pregnant women) ‘PE/HELLP’ (defined as previously described^43^), ‘prematurity’ (delivery <37 weeks of gestation), perinatal mortality (fetal loss <20 weeks of gestation or neonatal loss during first 28 days after delivery) and macrosomia (birth weight > 90th percentile corrected for gestational age, sex and parity^44^).

^*^Significantly different from the respective non-pregnant control, Independent T-test and Chi-square test, p < 0.05.

^†^Significantly different from the respective pregnant control, Independent T-test and Chi-square test p < 0.05.
